# B Chromosomes of the Asian Seabass (*Lates calcarifer*) Contribute to Genome Variations at the Level of Individuals and Populations

**DOI:** 10.3390/genes9100464

**Published:** 2018-09-20

**Authors:** Aleksey Komissarov, Shubha Vij, Andrey Yurchenko, Vladimir Trifonov, Natascha Thevasagayam, Jolly Saju, Prakki Sai Rama Sridatta, Kathiresan Purushothaman, Alexander Graphodatsky, László Orbán, Inna Kuznetsova

**Affiliations:** 1 Theodosius Dobzhansky Center for Genome Bioinformatics, Saint Petersburg State University, St. Petersburg 199004, Russia; andreyurch@gmail.com; 2 Reproductive Genomics Group, Temasek Life Sciences Laboratory, Singapore 117604, Singapore; shubha_vij@rp.edu.sg (S.V.); natmay@gmail.com (N.T.); jolly@tll.org.sg (J.S.); prakki_sr_sridatta@ttsh.com.sg (P.S.R.S.); kathiresan.purushothaman@nord.no (K.P.); 3 School of Applied Science, Republic Polytechnic 9 Woodlands Avenue 9, Singapore 738964, Singapore; 4 Institute of Biodiversity, Animal Health & Comparative Medicine, College of Medical, Veterinary & Life Sciences, University of Glasgow, Glasgow G12 8QQ, UK; 5 Institute of Molecular and Cellular Biology, Siberian Branch of the Russian Academy of Sciences, Novosibirsk 630090, Russia; vlad@mcb.nsc.ru (V.T.); graf@mcb.nsc.ru (A.G.); 6 Department of Natural Science, Novosibirsk State University, Novosibirsk 630090, Russia; 7 Faculty of Biosciences and Aquaculture, Nord University, 8049 Bodø, Norway; 8 Department of Animal Sciences, Georgikon Faculty, University of Pannonia, H-8360 Keszthely, Hungary; 9 Center for Comparative Genomics, Murdoch University, 6150 Murdoch, Australia

**Keywords:** teleost, population analysis, whole genome resequencing, DNA copy number variation, ribosomal DNA, B chromosomes

## Abstract

The Asian seabass (*Lates calcarifer*) is a bony fish from the Latidae family, which is widely distributed in the tropical Indo-West Pacific region. The karyotype of the Asian seabass contains 24 pairs of A chromosomes and a variable number of AT- and GC-rich B chromosomes (Bchrs or Bs). Dot-like shaped and nucleolus-associated AT-rich Bs were microdissected and sequenced earlier. Here we analyzed DNA fragments from Bs to determine their repeat and gene contents using the Asian seabass genome as a reference. Fragments of 75 genes, including an 18S rRNA gene, were found in the Bs; repeats represented 2% of the Bchr assembly. The 18S rDNA of the standard genome and Bs were similar and enriched with fragments of transposable elements. A higher nuclei DNA content in the male gonad and somatic tissue, compared to the female gonad, was demonstrated by flow cytometry. This variation in DNA content could be associated with the intra-individual variation in the number of Bs. A comparison between the copy number variation among the B-related fragments from whole genome resequencing data of Asian seabass individuals identified similar profiles between those from the South-East Asian/Philippines and Indian region but not the Australian ones. Our results suggest that Bs might cause variations in the genome among the individuals and populations of Asian seabass. A personalized copy number approach for segmental duplication detection offers a suitable tool for population-level analysis across specimens with low coverage genome sequencing.

## 1. Introduction

The Asian seabass is an economically important food fish species, the aquaculture production of which is rapidly spreading to a large number of countries extending beyond its native range [1,2]. It is a euryhaline and catadromous species with a wide geographic distribution stretching from Northern Australia to at least the Western part of India. A comprehensive analysis of the identity of the Asian seabass across its geographical range of distribution was described earlier using morphometric data and/or key mitochondrial sequences [3,4,5] and later based on whole genome resequencing information [6]. Sequence data of three mitochondrial markers (cytochrome c oxidase subunit 1 COI, 16S rDNA and D-loop) pointed to the existence of at least two distinct species, the first representing the Indian subcontinent, and the second South-East Asia (Singapore, Malaysia, Thailand, and Indonesia) together with Australia/Papua New Guinea, with the latter showing signs of splitting from the South-East Asian region [3,4,5,6].

The Asian seabass in Australia has a stronger migration capability than in South-East Asia and is adapted to both freshwater and marine environments [7]. The Asian seabass in South-East Asia shows an earlier maturation time than in Australia/Papua New Guinea [8] and was found only in marine water [9]. All Asian seabass populations were examined using 32,433 single-nucleotide polymorphisms (SNPs). [6]. It has revealed a genetic difference between South-East Asian and Australian/Papua New Guinean populations. There has been no recent gene flow between Asian seabass from South-East Asia and Australian/Papua New Guinea; however, the current patterns of genetic variability were likely caused by the co-effects of founder events, random genetic drift, mutations, and local selection [10]. The Asian seabass is a protandrous hermaphrodite with most individuals typically maturing as males at about a year of age and subsequently changing their sex to females [7,8]. Sex reversal in hermaphrodites may happen during a period of a few weeks, or up to several months [11,12,13]. The reproductive cycle of hermaphroditic teleosts can be affected by various environmental factors and is also known to be associated with the age of fish [11,14]. Comparative analysis of the expression of genes with a sex-associated function (e.g., germ cell markers, *Wnt* and retinoic acid signaling genes, and apoptotic genes) between the two gonad types showed differences in the patterns observed in mammals and other teleosts [13].

The Asian seabass genome was sequenced recently using long-read sequencing technology, followed by a multi-step assembly involving optical mapping and a comparative analysis of syntenic regions among three fish genomes, resulting in an assembly size of 670 Mb [6]. The diploid Asian seabass karyotype contains 24 pairs of A chromosomes and a variable number of either AT- or GC-rich B chromosomes (Bchrs or Bs in short) [6]. Bs, also known as supernumerary or accessory chromosomes, sometimes occur in eukaryotic genomes, typically constitute 1–5% of genome size and represent a non-essential and dynamic component of the genome. Accumulation of Bs may take place in either males or females and copy number polymorphism is often population-specific [15,16,17]. Bs are found in ~15% of karyotyped eukaryotic species and exhibit significant similarity in their morphology and structure in many species [15,18]. Certain common features have been demonstrated for Bs of various species, including plants. For instance, they have been shown to be comprised of an arrangement of A chromosome fragments [17,19,20]. The presence of Bs in the grass species *Aegilops speltoides* increases the frequency of heterologous synapses and recombination, causing new chromosomal abnormalities [21]. In addition, DNA fragments like rDNAs, tandem repeats, and clustered mobile elements have been documented in Bs of different plants [17,20,22,23], insects [24,25], fishes [7,23,24,26], and mammals [18,27,28]. These impact on the tissue-specific dynamics of mobile elements and tandem repeats [17,21,26]. The presence of genes on Bs was also demonstrated [17,18,19], however, the functional role and precise structure of Bs in most species are still unclear.

Previously, we isolated and sequenced fragments of 4′,6-diamidino-2-phenylindole (DAPI)-stained supernumerary chromosomes and presented their preliminary analysis, demonstrating homology of Asian seabass B-related fragments to four genomic scaffolds located on four specific linkage groups (LG5, LG9, LG17 and LG19) and several genomic regions that have not been assigned to the LGs [6]. In this study, we performed a more detailed cytogenetic and sequencing analysis of AT-rich Asian seabass Bs. In addition, we analyzed the content contribution of Bs to population-specific genomic changes among Asian seabass populations collected from India, South-East Asia/Philippines and Australia/Papua New Guinea.

## 2. Materials and Methods

### 2.1. Fish Material and DNA Extraction

Wild Asian seabass were procured from different locations. The freshly caught fishes were then obtained from the landing centers followed by sample collection. Farmed Asian seabass (Lates calcarifer) were obtained from the Marine Aquaculture Centre (Singapore). All experiments were approved by Agri-food and Veterinary Authority (AVA) Institutional Animal Care and Use Committee (IACUC) (approval ID: AVA-MAC-2012-02) and performed according to guidelines set by the National Advisory Committee on Laboratory Animal Research (NACLAR) for the care and use of animals for scientific research in Singapore.

To study the potential variation in the number of B-associated regions at a population scale, 50 wild-caught Asian seabass samples, collected from 13 geographic regions across the native range: India Western coast (four specimens) and Indian Eastern coast (five), South-East Asia (20), Philippines (four), Australia (12) and Papua New Guinea (five), were re-sequenced to ~7× average sequencing depth using the HiSeq 1500 Illumina platform as reported previously [6]. 

Asian seabass larvae at 1–2 days post-hatching (dph) were euthanized by placing them on ice, dissected and used for the culturing of primary fibroblasts and chromosome preparation as described previously [6,29]. In addition, we used small pieces of fins from three males and three females (nine months of age) for fibroblast culturing. Liver, skin, ovary and testis of ten male and female (nine-month-old) fish were used for DNA/RNA extractions as well as cell isolation for fluorescence-activated cell sorting (FACS) analysis. DNA was extracted using Qiagen kit (Qiagen, Hilden, Dermany) according to the manufacturer’s instructions. 

### 2.2. Estimation of DNA Content in Different Tissues by Flow Cytometry

Nuclei were extracted from male and female gonads, skin and livers as described previously [30]. The nuclei suspension was stained with propidium iodide (PI) for estimation of total nuclear DNA content [31]. The fluorescence peak of G0/G1 nuclei isolated from the liver of chicken (standard) was used as reference standard. For each sample, when possible, at least three independent replicate measurements were performed. Each probe contained 5000–10,000 nuclei, the average coefficient of variation for the probes and dyes ranged between 2–5%. The genome size was calculated by multiplying the size of the standard genome (chicken) with the ratio of their relative fluorescence intensities. The average 2C-value (in pg) was converted to bp size considering that 1 pg of DNA corresponds to 0.978 × 10^9^ bp [32]. A comparison between the individual data was performed using the Student *t*-test, *p* < 0.01.

### 2.3. Isolation and Sequencing of B Chromosomes 

B chromosome microdissection, amplification, library construction, sequencing and assembly have been described previously [6]. B chromosome-specific libraries (Bchr5 and Bchr6) were sequenced on a MiSeq genome sequencer (Illumina) in the Genomics Core Facility (Institute of Chemical Biology and Fundamental Medicine, Siberian Branch of the Russian Academy of Sciences, Novosibirsk, Russia). After trimming, MiSeq reads were then mapped to the Asian seabass reference genome assembly [6]. The consensus sequence motifs, obtained from the mapping of reads, were further chained together across increasing lengths of a spacer to form pseudo-scaffolds [6].

### 2.4. Fluorescence In Situ Hybridization (FISH) and Immunohistochemical Analysis

Isolation of Asian seabass *Cot-1* DNA was described in our earlier study [29]. The whole genome amplification (WGA1) PCR-amplified DNA of the micro-dissected B chromosomes and *Cot-1* were re-amplified in the presence of 16-dUTP-biotin and digoxigenin-11-dUTP, respectively (the final concentration of each was 2 μM, Roche (Basel, Switzerland) under the following conditions: (1×) 94 °C for 5 min; (35×) 90 °C for 30 s, 54 °C for 30 s, 72 °C for 30 s using a WGA3 reamplification kit (Sigma-Aldrich, St. Louis, MO, USA). Preparation of the 18S rDNA probe was performed by amplification from genomic DNA using specific primers 5′-GCGAAGGGTAGACACACGCTGA-3′, 5′-CCTCTAGCGGCACAATACGAATG-3′ [33].

The DNA mixture of ~400 ng of the Bchr-derived painting probe and 10 μg of the *Cot-1* fraction of the Asian seabass DNA was prepared. A fluorescent in situ hybridization (FISH) analysis was performed as described earlier [6,28]. Signal detection was accomplished using streptavidin conjugated with AlexaFluor-594 (ThermoFisher Scientific, Waltham, MA, USA) and anti-digoxigenin-fluorescein (Roche) to detect digoxigenin. 

After post-hybridization washes, an anti-5-MeC antibody (AbCam, Cambridge, UK) was applied. An anti-mouse AlexaFluor-488 antibody was used to detect the signal. Finally, the slides were counterstained with DAPI and mounted in an antifade solution (Vectashield from Vector laboratories, Burlingame, CA, USA).

Chromosome spreads were incubated with rabbit a centromere-specific protein A (CENP-A) antibody (Aviva Systems Biology, San Diego, CA, USA) in blocking solution overnight at 4 °C, followed by a 1 h room temperature incubation with a secondary biotinylated anti-rabbit antibody (Vector Lab, Peterborough, UK).

Chromosomal images were captured on a Zeiss/MetaMorph epifluorescence microscope equipped with a Nikon (CCD) camera. Fibroblast sections were examined using a Zeiss LSM 510 META inverted microscope. Images were analyzed using Image-Pro Express software V5.0 (Media Cybernetics, CA, USA). The path of the chromosomes was computationally traced and straightened according to the manual provided by the Image J software V1.41 (http://rsb.info.nih.gov/ij).

### 2.5. Annotation of B Chromosome-Derived Pseudo-Scaffolds

Gene descriptions were assigned to the B-derived pseudo-scaffolds based on gene prediction and annotation of the corresponding regions in the Asian seabass genome [6] and Zebrafish Model Organism Database (ZFIN) [34]. Repeat analysis was performed using the Asian seabass repeats database [6] and the Repbase dataset for vertebrate genomes (http://www.girinst.org/repbase/).

### 2.6. Data Availability

The scaffolded genome assembly has been submitted to DDBJ/EMBL/NCBI GenBank under the accession number of LLXD00000000.1. Alternatively, it is also available for download at http://seabass.sanbi.ac.za/. The Illumina and PacBio reads utilized for the genome assembly, as well as the whole-genome resequencing reads, have been submitted to NCBI SRA under BioProject accession numbers SRP069219 and SRP069848, respectively. The raw reads obtained from the sequencing of Bchr5 and Bchr6 libraries have been deposited in the NCBI SRA database under the BioProject accession number SRP082620.

### 2.7. Population-Level Analyses of Asian Seabass B Chromosomes from Re-Sequencing Data

Population scale variation in the number of copies of B-associated regions was estimated using the read-depth based algorithm [35]. Trimmed reads from the 51 sequenced samples, including the reference genome, were aligned to the repeat-masked Asian seabass reference genome (version 2.0) using Bowtie2 [36] with -a mode (search for and report all alignments). Read alignment and SNP-calling in repeats are unreliable because of the high misalignment rate and their problematic assembly; thus, repeat-rich regions were excluded from this analysis. The resulting sequences alignment map (SAM) files were processed using mrCaNaVaR [35] to produce a genome-wide GC-corrected copy number of the genome fragments in 1000 bp windows, excluding repetitive and gapped regions of the genome. A copy number variation analysis was performed using the samples with the most uniform coverage to achieve comparativeness between the studied individuals from the previous study [6].

Two single-exon genes were selected as controls as described earlier [37]: ZNFX1-type zinc finger-containing protein 1-like *(znfx-1)* and galactose-3-*O*-sulfotransferase 3 *(gal3st3)*. The control genome fragment with a size similar to that of Bchr6 (~400 kb) was taken from LG20 (unitig_2 from 6205808 to 6605807). This fragment did not have any sequences similar to Bchr6.

### 2.8. Estimation of the Density of Single Nucleotide Variants and Polymorphisms

Estimation of the density of single nucleotide variants and polymorphisms (SNVs/SNPs) in the B-associated regions was performed based on SNP-calling results from the Asian seabass resequencing data published earlier [6]. SNP-calling was carried out using the Samtools pipeline [6]. The corresponding coordinates for the 10 kb spacer B-derived pseudo-scaffolds were extracted from the whole genome SNP-calling results. Possible functional effects of SNVs in the B regions were evaluated using SNPeff [38].

### 2.9. Expression of Predicted B-Chromosomal Genes

Information for the expression of genes identified from B chromosomal pseudo-scaffolds was obtained from microarray-based data that was previously produced for the analysis of the Asian seabass gonadal transcriptome [39]. 

## 3. Results

### 3.1. Asian Seabass B Chromosomes are Repeat-Rich and Associated with Nucleolus

**Figure 1:**
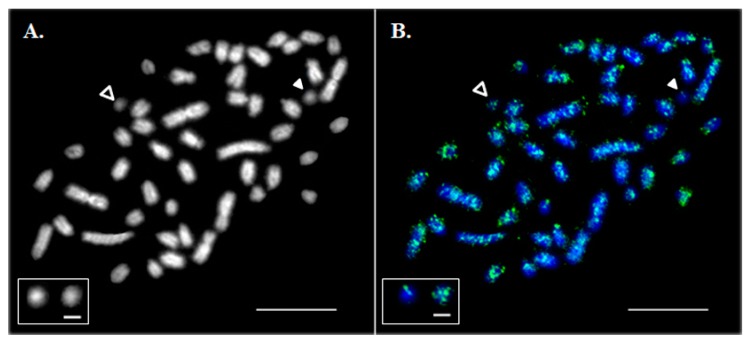
Hybridization of mixed *Cot-1* DNA from liver, ovary and testis on metaphase chromosomes. (**A**) A DAPI-stained metaphase spread. (**B**) Asian seabass *Cot-1* DNA fraction hybridization (green). Chromosomes were contrasted by 4′,6-diamidino-2-phenylindole (DAPI; blue). Empty arrowheads indicate B chromosomes (Bchrs). White arrowheads indicate Bchrs with a low level of *Cot-1* signal. The bar is 5 μm. The inset shows magnified examples of Bchrs with a low (left) and high (right) intensity of *Cot-1* signal; the bar for the framed B chromosomes is 1 µm.

Dot-like Bs were estimated to constitute 0.05–0.5% of the DAPI-stained area of all metaphase chromosomes (Figure 1A). The number of DAPI-stained Bs in the primary fibroblast cell culture did not show any specific association with sex and varied from zero to four across individual metaphase spreads, suggesting an intra-individual mosaicism.

To examine the contribution of Bs to DNA content differences between certain tissues, we estimated the DNA content in liver or skin as well as male and female gonads using a flow cytometric approach. The data showed that the diploid DNA content in the ovary of the mature Asian seabass was significantly lower than that of the testis (705 ± 42 Mb vs. 773 ± 50 Mb; *n* = 10 per sex; *p* < 0.01; Student’s *t*-test). On the other hand, there was no statistical difference in genome size between male and female hepatocyte or skin nuclei (734 ± 66 Mb vs. 747 ± 54 Mb; *p* < 0.01; *n* = 10 per sex; Student’s *t*-test).

In situ hybridization of a labelled Asian seabass *Cot-1* DNA probe to its own chromosomes revealed that repetitive sequences were abundant in the genome. Their distribution was dispersed along the length of all A chromosomes with an insignificant accumulation on the centromeric and telomeric regions whereas some of the Bs appeared to be largely free from *Cot-1* DNA hybridization signals (Figure 1B).

**Figure 2:**
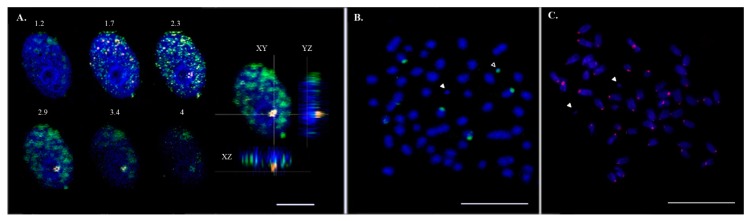
Micro-dissected B chromosome-derived probes associated with the nucleolus and 18S rDNA were detected on both A and B chromosomes of the Asian seabass. (**A**) Hybridization of Bchr6 painting probes on primary fibroblast cell nuclei. Confocal section with a 0.5 μm step and three nuclear projections (XY, YZ, XZ). Micro-dissected probes of Bchr6 (red), anti-5-methylcytosine AB (green). Nuclei were counterstained with DAPI (blue). The bar is 5 μm. (**B**) B chromosomes with and without 18S rDNA (green signal). White arrowheads indicate a Bchr without 18S rDNA; empty arrowheads indicate a Bchr with an 18S rDNA signal. The bar is 5 μm. (**C**) Histone H3-like centromere protein A (CENP-A) stained chromosomes (red signal). White arrowheads indicate a Bchr without CENPA. The bar is 5 μm.

We have shown earlier that fluorescent in situ hybridization signals of Bchr5- and Bchr6-derived painting probes result in an overlapping pattern [6]. In the interphase nuclei, Bchr-derived, labeled probes occupied a highly methylated region, indicating that the chromatin of Bchrs was not active at this stage and was in contact with the nucleolus (Figure 2A). Two pairs of autosomes with an 18S rDNA signal were identified on the metaphase spread. Bchrs with and without 18S rDNA are shown in Figure 2B. The presence of the centromere/kinetochore protein A (CENP-A) could not be detected on Bchrs (Figure 2C).

### 3.2. B Chromosomal Repeats 

The assembly size of Bchr5 and Bchr6, together with the 10 kb spacer, was 386,725 bp, representing ~0.067% of the Asian seabass reference genome assembly [6]. This is similar to the cytogenetic estimation of the total size of Bs. Therefore, subsequent downstream analyses were performed on the 10 kb-joined pseudo-scaffolds. 

Here we demonstrated that the Bchr6 assembly contained just ~2% of characterized repeats compared to 16.75% present in the whole genome. Short fragments of transposons were identified in both assemblies of Bs (Table S1). In spite of FISH signal accumulation for the Bchr6 and Bchr5 probes in the centromeric region of metaphase A chromosomes. We did not find any sign of the centromeric/pericentromeric satellite DNA of A chromosomes within the Bchrs libraries (Table S1). 

**Figure 3:**
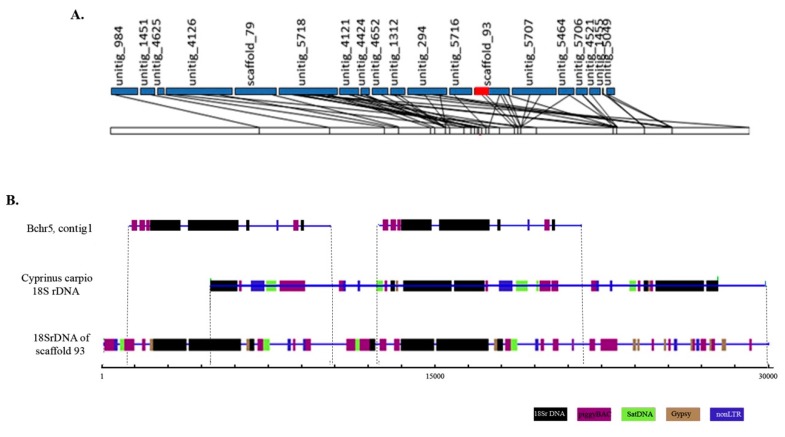
The 18S rDNA region of B chromosome 5 (Bchr5) shows a complex arrangement of various fragments of transposable elements with ribosomal genes and is different from the 18S rDNA region of the Asian seabass autosomal chromosomes. (**A**) Diagrammatic representation of genetic linkage group 5 (LG5). The ribosomal region located on genomic scaffold_93 assigned to LG5 is labeled in red. (**B**) Alignment of ribosomal genes containing the region of scaffold_93 of the Asian seabass genome, contig 1 of micro-dissected Bchr5 and rRNA sequence of *Cyprinus carpio* (9426 bp; JN628435). Various fragments of repetitive elements are indicated as follows: Satellite DNA (green), piggy BAC and R2 (violet), Gypsy (brown), and 18S rDNA (black).

Bchr5-specific sequences contained a fragment homologous to LG5 as well as fragments of the Asian seabass genome without any linkage group assignment [6]. In the Asian seabass genome assembly, one of the sequences coding for rDNAs was located as a repeat unit on scaffold_93 assigned to LG5 (Figure 3A). The Bchr5 assembly contained short fragments of unknown DNA and a 24,841 bp contig (contig 1), which aligned with a major ribosomal rRNA region. Three copies of 18S rDNA were found in contig 1 of Bchr5 (Figure 3B, Table S1). The 18S rRNA of Bchr5, as well as scaffold 93, of the Asian seabass genome, was compared with the *Cyprinus carpio* 18S rRNA fragment (JN628435, 9426 bp, RefSeq). The results showed that the 18S ribosomal RNA genes and internal transcribed spacer 1 for both genomes and Bchr5 were similar but not identical (Figure 3B). The 18S rDNA region from the Asian seabass genome assembly (scaffold 93) and Bchr5 assembly showed a complex arrangement of 18S rDNA and various fragments of transposable elements, mainly from the piggyBAC and Gypsy families (Figure 3, Table S1).

### 3.3. B chromosomal Genes

In total, 75 genes were identified in the Bchr6 assembly (listed in Table S2), whereas just 18S rDNA was identified from Bchr5. The average gene densities for the assembled genome and the partial Bchrs assembly were 30.2 per kb and 5.16 per kb respectively. Thus, six times more genes per kilobase were present in the assembled genome compared to the Bchr6 assembly. On the other hand, the density of SNVs in the B chromosomal regions was approximately eight per kb (2901 SNVs per 361,036 kb), a value identical to that of the Asian seabass genome (5,642,327 SNVs per 670 Mb; [6]). Genomic fragments representing Bchr6 showed different genomic structural variations including insertions and deletions, however, genomic structure variations such as splice-site disruption and a gain of stop codons could not be identified. No specific function could be attributed to ~30 of the annotated B-specific genes (Table S2), while another ~30 genes were found to be expressed in most of the organs (Table S2, ZFIN [6]). The remaining ten genes were found to have a specific expression associated with the gonads and the brain (*astn2*, *bre*, *dpf3*, *fbxo33*, *fkbp*, *gabrb2*, *myt1l*, *rab14*, *rxrab*, and *pacrg*, see Table S2).

### 3.4. Population-Level Analysis of B Chromosome-Associated Regions Supports the Existence of an Asian Seabass Species Complex 

**Figure 4:**
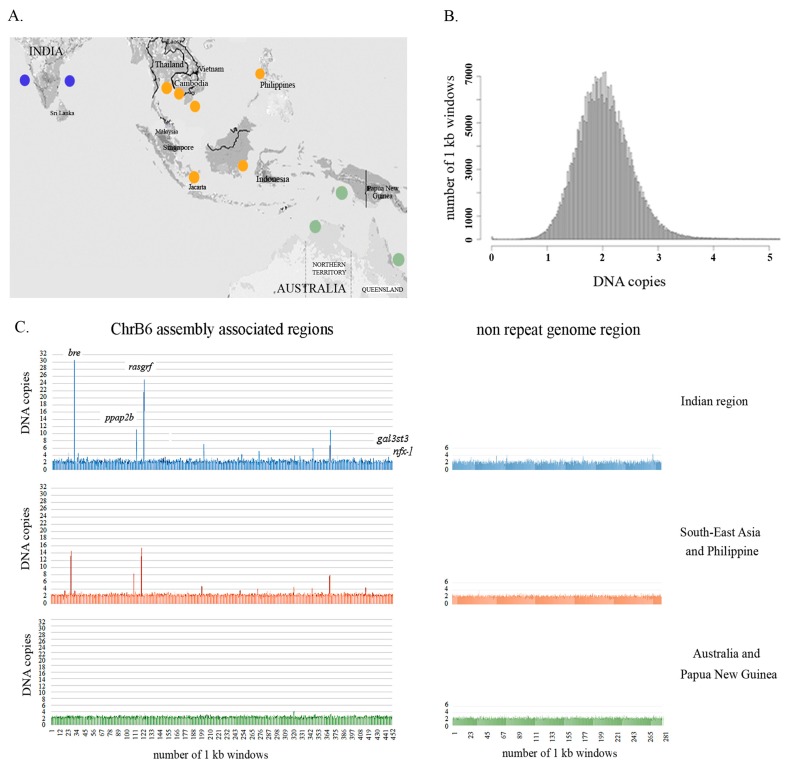
Population-scale variation in the copy number of B chromosome-associated regions in the Asian seabass species complex. (**A**) Geographic regions across the native range of Asian seabass: Western and Eastern Coast of India; South-East Asia and the Philippines, Australia/Papua New Guinea. (**B**) Distribution of inferred genome fragment copy number in 1 kb windows across the Asian seabass genome: Homozygous deletion, 0.1–1.4; heterozygous deletion, 1.5–2.5; normal diploid copy number, 2.6–4; heterozygous duplication, > 4; multiple duplications. X-axis: DNA copies; Y-axis: Number of 1 kb windows. (**C**) Population-scale variations in the copy number of the Bchr6-associated region and control genome fragment were demonstrated for the three geographical regions. X-axis: Number of genomic fragments which correspond to a particular 1 kb window of the reference genome for each individual. Y-axis: Genomic fragment copy number in 1 kb windows across the Bchr6-associated region and non-repeat genomic fragment. The order is indicated in Table S3: Indian region (blue), South-East Asia and Philippine region (orange), Australia/Papua New Guinea region (green). The copy number of control genes (*gal3st3*, *znfx-1*) is presented in 442–452 regions. The estimation of copy number variation was performed after repeat masking, so the number of DNA fragments and size of the analyzed sequences were less than the source. The Asian seabass genome assembly version 2.0 was used as a reference genome.

We have performed a detailed analysis of the copy number variation of Bchr6, whereas Bchr5 was excluded from this analysis due to the enrichment of repeats (Figure 3, Table S1). Analyses of sequences representing Bchr6 from the 51 individuals across the different geographical regions (Figure 4A) revealed copy number variations in 438 unique genomic fragments (Table S3). Of these, 198 were annotated while 240 were not described earlier as genes or repeats. DNA fragments representing Bchr6 were observed to have a tendency to multiply (>3 copies per haploid genome) in the genomes (Figure 4C; Table S3). The copy number variation profile amongst the Bchr6 fragments was more variable in the Indian seabass individuals compared to the other two populations. Individuals from Australia and Papua New Guinea had minimal copy number variation (Figure 4C; Table S3).

Samples from the Indian and South-East Asia/Philippines regions had a wider range of copy number variation between specimens compared to those from Australia and Papua New Guinea. A wide range of copy numbers from Indian and South-East Asia/Philippines specimens was detected for gene fragments coding for brain and reproductive organ-expressed proteins (*bre*; copy number range 1.1–30.6 and 1.2–14.6 for India and South-East Asia respectively), phosphatidic acid phosphatase type 2B (*ppap2b*; 1.1–11.2 vs. 1.5–8.25), and Ras protein-specific guanine nucleotide-releasing factor 1 (*rasgrf*; 1.6–25 vs.1.6–15.5) (Table S3, Sheet 1). Fragments corresponding to control genes (*gal3st3* and *znfx-1*, see scaffold 14 and unitig_4699 on Sheet 1 of Table S3) and the control genome fragment corresponding to fragment of LG20 (unitig_2, 6205808 to 6605807; see Sheet 2 of Table S3) had copy numbers typical for normal diploid genomes (1.5–3) for each of the 51 specimens from all three geographical regions (Figure 4C, Table S3, Sheet 2).

Thus, the data demonstrated a high level of polymorphism of the Bchr6 content providing further evidence and additional insight into the structuring of the three major geographical groups, which were shown earlier based on mitochondrial and nuclear markers, morphometric data and whole genome re-sequencing [5]. 

## 4. Discussion

At the time of its publication, the genome of the Asian seabass had the best assembly metrics among the de novo assembled fish genomes available thus far [6], which made it a good tool for detailed genomic and cytogenetic studies. The species is a protandrous hermaphrodite: Individuals typically mature as males and later reverse their sex to become females. The standard diploid chromosome number for male and female Asian seabass is identical (2*n* = 48), as demonstrated by the high-resolution linkage map constructed using 790 microsatellites and SNPs [40] and confirmed by cytological analysis [29]. 

Variable numbers of both AT- and GC-rich Bs were detected in metaphase plates of cultured primary fibroblast cells from adult male and female skin, as well as larvae of the Asian seabass [29]. Some metaphase plates of the Asian seabass fibroblast culture did not have any Bs, which points to an intra-individual mosaicism related to their unstable transmission during mitosis. The variability in the number of the Bs is associated with their typical non-Mendelian inheritance pattern and could be explained by their elimination during mitosis, possibly under the influence of environmental factors [15].

The presence of Bs usually increases the individual’s genome size but does not substantially affect the variability within the complex or population, regardless of whether individuals with accessory chromosomes are included [41]. Comparative sequence analyses of the genomes of B-carrying and B-lacking individuals from closely related populations are informative [20,24]. The inter-individual difference between the Asian seabass from various geographical regions was obvious (Figure 4). Investigation of the Asian seabass B-related fragments identified 438 DNA fragments with a tendency for amplifications or deletions (Table S3) in two of the three geographical regions of Asian seabass distribution, namely in India and South-East Asia/Philippines (Figure 4). Control genomic regions were slightly more variable in the Indian specimens than in those from South-East Asia/Philippines and Australia. Therefore, a more stable genome with less population fluctuation was demonstrated for the Australia/Papua New Guinea population. Thus, the copy number variation of the B-related fragments was in agreement with the deep phylogenetic analysis of the Asian seabass populations based on mtDNA markers, re-sequenced genomes [5,6] and SNP analyses [10]. Here, we have demonstrated the genetic diversity and patterns of population differentiation across all of the populations and we have provided significant evidence for genetic differentiation between South-East Asian and Australian/Papua New Guinean populations; in agreement with the results of Wang [10], who assumed that the Asian seabass of these regions had been evolving towards allopatric speciation since the split from the ancestral population during the mid-Pleistocene.

To summarize, the population analysis of the widely distributed Asian seabass has given an indication that supernumerary chromosomes could be involved in genome size variations between populations. The presence or absence of Bs in different populations of the Asian seabass can be linked to environmental factors in different geographical regions.

As described earlier, the sequenced heterochromatin from Asian seabass Bs (Bchr5 and Bchr6) did not contain any pericentromeric or centromeric sequences from autosomes [5,6,29]. In addition, the Bchr-derived repeats also had short fragments of transposable elements (TE) from the host genome (Table S1). This agrees with the cytogenetic examination of the repetitive DNAs (*Cot-1*) distribution on Bs, which was extremely weakly stained by the total repetitive DNA probe as well as with the absence of histone H3-like centromere protein A (CENPA) signals on Bs (Figure 2C). The centromeres of the rye Bs evolved from a standard centromere and lack sequences connected with centromeric, i.e., CENH3-histone-containing nucleosomes or histone H3-like centromeric protein A. Additional repeats accumulated in the centromere of the newly formed B chromosome [17,42]. A similar structure of a centromere may be characteristic for the Asian seabass Bs.

These results suggest that the repetitive DNA of the Asian seabass Bs had a specific set of repeats different from those of autosomal origin and might possess a specific centromere and sub-telomeric blocks (Figure 1B). Full-size transposons were absent from the Bchr5 and Bchr6 assemblies, however, transposon-derived fragments can maintain their functional activity in the genome. The role of particular repetitive elements and their fragments associated with the Bs of teleosts has also been actively discussed [24,41]. Multiplication of transposable element (TE) fragments in Bs of cichlid [20,42,43] and cyprinid [26] fish led to the proposal that TEs are responsible for the insertion of sequences into Bs [17,41]. This mechanism could explain the presence of fragments of different genes with a variable copy number. For example, Bs of the grasshopper *Eyprepocnemis plorans* contained large amounts of rDNA [42], whereas the arrangement of the ribosomal genes and surrounding rDNA-specific TEs, such as R2, Gypsy, RTE and mariner transposable elements is different in Bs and host genomes [18,20,24,25]. It was shown that R2 elements are transcribed in the ovaries and eggs but not in male tissues or embryos and different R2 5′ truncation patterns are found in natural populations, providing evidence for recent retro-transposition activity [24]. A similar organization was demonstrated for rRNA genes in Bs of *Drosophila virilis* [44], grasshopper *E. plorans* [24,25], and teleosts [45,46]. We observed that the Asian seabass Bs are located adjacent to the nucleolus within the nucleus, suggesting their potential contribution to nuclear formation (Figure 2 and Figure 3). The Asian seabass Bchr5 assembly contained fragments of 18S rRNA genes and short R2 fragments in addition to other elements (Table S1). Therefore, the repeat units of the rRNA genes are usually located very close to blocks of constitutive heterochromatin and they are flanked by transposon elements (Figure 3B). 

A comparison of Asian seabass Bs with those from different species revealed common features of these genomic elements. Bs typically contain thousands of sequences duplicated from the A chromosomes [18,42]. Most of the genes that originate from Bs are fragmented but some, including those influencing cell metabolism [47] and rRNA genes [48], may still maintain their transcriptional activity. Recently, a novel male-specific sequence, called *setdm* located on extra micro-chromosomes, was described in Gibel carp [49]. Fragments of genes, which are potentially located in Bs, did not contain any nonsense or missense mutations (see Material and Methods) and could be involved in different processes, including sexual development, cell division, immune response, and DNA repair (Table S2). 

Cytogenetic analysis of four Japanese hagfish species from the order Myxinida (*Eptatretus okinoseanus*, *Eptatretus burgeri*, *Paramyxine atami*, and *Myxine garmani*) revealed differences in the B numbers between germ cells (spermatocytes and spermatogonia) and somatic cells (liver, blood, gill, and kidney) [50]. The C-banding of metaphase chromosome preparations of germline and somatic cells from each hagfish species revealed that the C-band-positive chromatin in the ancestral somatic cells had been almost completely eliminated [51]. 

Microarray analysis of the expression of 60,080 Asian seabass genes was used earlier to identify differentially expressed genes in transforming gonad types that are in a sequential order of development [39]. From 75 genes identified on the Bchr6-derived pseudo-scaffolds, 42 were inventoried for differential expression in transforming gonads from the above-mentioned microarray experiment (Table S2). Therefore, we can assume that these 42 genes are presumably active in Bchr6 but final confirmation of their activity would require further experiments.

A number of different sex determination systems have been described for fishes [14,16,52,53]. They can even be different between populations of the same species. For example, a comparative analysis of the *Eigenmannia* genus suggests that their sex chromosomes may have arisen independently in the different populations [52,53]. A connection between Bs and sex determination was described for Amazon molly [54], in Lake Victoria cichlids [55] and some cyprinids [49]. A cytogenetic analysis of four Japanese hagfish species from the order Myxinida revealed differences in the B numbers between male germ cells (spermatocytes and spermatogonia) and somatic cells (liver, blood, gill, and kidney) [49]. 

In conclusion, Bs are a major source of intraspecific variation in the nuclear DNA amounts in numerous species of plants and create polymorphisms for DNA variation in natural populations [22]. Different populations of the Asian seabass may differ in the number and composition of B chromosomes. A personalized copy number approach for segmental duplication detection offers a suitable tool for a population-level analysis across specimens with low coverage genome sequencing.

## Supplementary Materials

The following are available online at http://www.mdpi.com/2073-4425/9/10/464/s1, Table S1: Repeats of Bchr5 assembly (Sheet 1), Bchr6 assembly (Sheet 2), and Asian seabass genome scaffold_93 for control (Sheet 3).; Table S2: The list of 75 genes predicted from the B chromosome 6 assembly and two single exon genes, which were used as controls; Table S3: Population-scale variation in the copy number of B chromosome 6-associated regions (Sheet 1) and control fragment of the Asian seabass reference genome (Sheet 2, unitig_2).
